# New Insights in the Research on Bioactive Compounds from Plant Origins with Nutraceutical and Pharmaceutical Potential II

**DOI:** 10.3390/plants14040500

**Published:** 2025-02-07

**Authors:** Ivayla Dincheva, Ilian Badjakov, Bistra Galunska

**Affiliations:** 1 Department of Agrobiotechnologies, Agrobioinstitute, Agricultural Academy, 8 Dragan Tsankov Blvd., 1164 Sofia, Bulgaria; ibadjakov@abi.bg; 2 Publishing Department, Medical University “Prof. Dr. Paraskev Stoyanov”, 84 Tzar Osvoboditel Str., 9000 Varna, Bulgaria; galunska@mu-varna.bg

Exploring bioactive compounds derived from plants has become a cornerstone of innovation in the nutraceutical and pharmaceutical sectors. Throughout history, plants have been integral to traditional medicine, serving as primary sources for healing and health promotion in countless cultures. These natural compounds encompassing alkaloids, terpenoids, and flavonoids, possess a wide array of health-promoting properties. They are lauded for their antioxidant, anti-inflammatory, antimicrobial, anticancer, and metabolic regulatory effects, making them critical to combating chronic and acute diseases. In recent decades, the increasing prevalence of lifestyle-related health issues such as obesity, diabetes, cardiovascular diseases, and cancer has intensified the search for natural and sustainable therapeutic alternatives. The limitations of synthetic drugs, including side effects, high costs, and drug resistance, have further bolstered the appeal of plant-based solutions. Bioactive compounds extracted from plants offer a holistic approach, addressing not just the symptoms, but also the underlying mechanisms of disease, through multiple biochemical pathways. Moreover, they align with the growing consumer demand for natural, minimally processed, and eco-friendly health solutions. Integrating plant bioactive components into nutraceutical and pharmaceutical products also reflects the broader scientific recognition of the link between diet and health.

This Special Issue explores the wide-ranging progress in studying plant-derived bioactive compounds. It focuses on their therapeutic and functional properties, examining how these compounds can benefit human health. Furthermore, it tackles significant challenges, such as fluctuations in compound yields driven by environmental and ecological conditions, as well as the demand for effective and scalable production solutions. This issue highlights the integration of analytical methods, computational tools, and biotechnological innovations with insights from ethnobotanical practices, offering a comprehensive perspective on the multifaceted role of these natural compounds in tackling health and environmental challenges.

A central theme of this collection is exploring the precise mechanisms through which plant-derived compounds exert their effects. For example, gintonin, a glycolipoprotein obtained from ginseng, has been demonstrated to suppress epithelial–mesenchymal transition (EMT), a vital process in cancer metastasis. It shows a strong anticancer potential by targeting the TGF-β signaling pathway and decreasing the phosphorylation of Smad2 and Smad3. Its precise molecular action provides a compelling basis for further exploration in pharmaceutical applications [1]. Similarly, eugenol found in aromatic plants exhibits strong antiproliferative effects on osteosarcoma and oropharyngeal cancer cells. Studies reveal that eugenol induces apoptosis by upregulating pro-apoptotic marker genes, such as Bax and Bad, while activating caspases that drive cell death. These findings position eugenol as a promising candidate for targeted cancer therapies, though challenges related to its delivery and bioavailability remain [2]. The potential of computational tools in advancing plant bioactive research is exemplified by studies on *Perilla frutescens*. Network pharmacology revealed the multi-targeted activity of rosmarinic acid, the primary metabolite in ethanol leaf extracts, against prostate cancer. By mapping its interactions with cancer-related pathways, researchers identified key regulatory nodes in gene networks that mediate its effects. This approach not only elucidates the molecular basis of its therapeutic potential, but also offers a framework for optimizing plant extracts for multifunctional applications [3].

Beyond cancer therapeutics, plant-derived compounds have demonstrated significant roles in regulating metabolic health. Research into α-amylase inhibitors highlights their ability to reduce the hydrolysis of dietary starch, providing an effective strategy for managing type 2 diabetes by lowering postprandial glucose levels. These findings underscore the potential of these inhibitors to address global health challenges, though scaling their production remains an ongoing obstacle that demands the exploration of sustainable methodologies [4].

Essential oils from *Ocimum* species offer a compelling example of plant-derived substances with antimicrobial and anti-inflammatory properties. Rich in bioactive constituents such as estragole, thymol, linalool, and eugenol, their composition is shaped by genetic diversity and environmental factors. These essential oils demonstrate a multifaceted utility in both food and pharmaceutical applications by interacting with microbial and immune pathways [5].

The bioactivity of plant-derived substances is influenced by factors such as environmental and ecological conditions. Studies on cranberry (*Vaccinium oxycoccos*) reveal substantial variations in anthocyanin and flavonol composition depending on habitat-specific factors, such as oligotrophic versus mesotrophic wetlands and seasonal harvesting periods. These findings highlight the importance of targeted cultivation strategies to optimize bioactive yields while preserving biodiversity [6]. Similarly, extracts of *Frangula alnus* Mill. demonstrate dual benefits, supporting gut health by remaining non-disruptive to probiotic bacteria such as *Lactobacillus rhamnosus*, while altering bacterial membrane permeability to regulate cellular transport. This suggests potential applications in enhancing the efficacy of other bioactive substances [7].

Sustainability and biodiversity emerge as critical considerations in bioprospecting for therapeutic applications. Research on *Sloanea chocoana* and *S. pittieriana* exemplifies this balance, using GC-MS techniques to identify high-value compounds such as polyphenolics, triterpenoids, and alkaloids. These compounds contribute to antioxidant, antifungal, and photoprotective activities, underscoring their application in cosmeceutical products while emphasizing the need for sustainable harvesting practices to preserve ecological integrity [8].

Traditional knowledge remains a guiding force in exploring plant-derived compounds, as illustrated by the study of *Verbena officinalis*. Biochemical assays and histological analyses validated its protective effects against oxidative stress by modulating antioxidant enzymes such as superoxide dismutase and glutathione peroxidase, highlighting its potential for mitigating stress-induced physiological damage [9]. Similarly, metabolite profiling of peach (*Prunus persica*) varieties employed HS-SPME-GC-MS and multivariable statistical analysis to characterize volatile compositions and metabolic profiles, emphasizing the role of fruit skin in flavor optimization and bioactive richness. Though integrating by-products into functional food development, these studies link traditional agricultural practices with contemporary innovations [10].

Innovative methodologies continue to transform the study and application of plant-based compounds. The elicited cell cultures of tamarillo (*Solanum betaceum*) represent a breakthrough in enzyme production. Using biotic elicitors like casein hydrolysate and chitosan, researchers enhanced the production of hydrolytic enzymes, positioning tamarillo as a scalable plant-based bioreactor system for industrial and pharmaceutical applications [11]. Building on the theme of therapeutic innovation, latex extracts from *Euphorbia seguieriana* and *E. cyparissias* reveal significant anticancer potential by inhibiting P-glycoprotein (P-gp) function in multidrug-resistant cancer cells. These findings highlight their promise as modulators of drug resistance for novel cancer therapies [12].

Nutritional and therapeutic contributions from *Pereskia* sp. (*Ora-Pro-Nobis*), rich in proteins, fiber, and phenolic compounds, demonstrate its dual utility as a dietary resource and a source of bioactive compounds with antioxidant and anti-inflammatory properties. These characteristics underscore its significance in both traditional and functional foods [13]. Similarly, transdermal delivery systems enriched with plant-derived compounds, such as hydrogels and microneedles, exemplify advances in wound healing and controlled drug release by enhancing bioavailability and stability [14].

Finally, *Moringa oleifera* illustrates the versatility of plant-based substances. Its leaves, rich in flavonoids, tannins, and phenolic acids, exhibit antiparasitic efficacy against gastrointestinal nematodes, highlighting its dual role as a nutraceutical and a natural veterinary remedy [15]. The research on *Pistacia lentiscus* further emphasizes its polyphenol-rich extracts, which display antioxidant, anti-inflammatory, antimicrobial, and anticancer properties. These effects, influenced by the plant’s geographic origin and phenological stage, underline its potential for food and pharmaceutical applications [16].

The studies in this Special Issue collectively demonstrate the convergence of traditional knowledge, cutting-edge science, and interdisciplinary collaboration in unlocking the potential of plant-derived compounds. By addressing global health challenges, enhancing functional food and pharmaceutical applications, and promoting sustainable practices; these contributions lay a solid foundation for future advancements. As we move forward, integrating advanced technologies with ecological stewardship will be essential in shaping a future where plant-derived substances achieve their fullest potential for human and environmental well-being.

## References

[1] Kim S.J., Nah S.-Y., Park I.-H., Shin M.-S., Kang K.S. (2023). Gintonin Isolated from Ginseng Inhibits the Epithelial-Mesenchymal Transition Induced by TGF-β in A549 Lung Cancer Cells. Plants.

[2] Racea R.-C., Macasoi I.-G., Dinu S., Pinzaru I., Marcovici I., Dehelean C., Rusu L.-C., Chioran D., Rivis M., Buzatu R. (2023). Eugenol: In Vitro and In Ovo Assessment to Explore Cytotoxic Effects on Osteosarcoma and Oropharyngeal Cancer Cells. Plants.

[3] Garcia P.J.B., Huang S.K.-H., De Castro-Cruz K.A., Leron R.B., Tsai P.-W. (2023). An In Vitro Evaluation and Network Pharmacology Analysis of Prospective Anti-Prostate Cancer Activity from Perilla frutescens. Plants.

[4] Kashtoh H., Baek K.-H. (2023). New Insights into the Latest Advancement in α-Amylase Inhibitors of Plant Origin with Anti-Diabetic Effects. Plants.

[5] Mulugeta S.M., Pluhár Z., Radácsi P. (2024). Phenotypic Variations and Bioactive Constituents among Selected *Ocimum* Species. Plants.

[6] Šedbarė R., Sprainaitytė S., Baublys G., Viskelis J., Janulis V. (2023). Phytochemical Composition of Cranberry (*Vaccinium oxycoccos* L.) Fruits Growing in Protected Areas of Lithuania. Plants.

[7] Kledecka A., Siejak P., Pratap-Singh A., Kowalczewski P.Ł., Fathordoobady F., Jarzębski M., Smułek W. (2022). Extracts from *Frangula alnus* Mill. and Their Effects on Environmental and Probiotic Bacteria. Plants.

[8] Quintero-Rincón P., Pino-Benítez N., Galeano E., Rojo-Uribe C., Mesa-Arango A.C., Flórez-Acosta O.A. (2023). *Sloanea chocoana* and *S. pittieriana* (Elaeocarpaceae): Chemical and Biological Studies of Ethanolic Extracts and Skincare Properties. Plants.

[9] Rodrigues Oliveira S.M., Dias E., Girol A.P., Silva H., Pereira M.d.L. (2022). Exercise Training and *Verbena officinalis* L. Affect Pre-Clinical and Histological Parameters. Plants.

[10] Mihaylova D., Popova A., Dincheva I. (2022). Pattern Recognition of Varieties of Peach Fruit and Pulp from Their Volatile Components and Metabolic Profile Using HS-SPME-GC/MS Combined with Multivariable Statistical Analysis. Plants.

[11] Casimiro B., Mota I., Veríssimo P., Canhoto J., Correia S. (2023). Enhancing the Production of Hydrolytic Enzymes in Elicited Tamarillo (*Solanum betaceum* Cav.) Cell Suspension Cultures. Plants.

[12] Jadranin M., Savić D., Lupšić E., Podolski-Renić A., Pešić M., Tešević V., Milosavljević S., Krstić G. (2023). LC-ESI QToF MS Non-Targeted Screening of Latex Extracts of *Euphorbia seguieriana* ssp. *seguieriana* Necker and *Euphorbia cyparissias* and Determination of Their Potential Anticancer Activity. Plants.

[13] Teixeira V.M.C., Oliveira A.d., Backes E., Souza C.G.M.d., Castoldi R., Sá-Nakanishi A.B.d., Bracht L., Comar J.F., Corrêa R.C.G., Leimann F.V. (2023). A Critical Appraisal of the Most Recent Investigations on Ora-Pro-Nobis (*Pereskia* sp.): Economical, Botanical, Phytochemical, Nutritional, and Ethnopharmacological Aspects. Plants.

[14] Isopencu G.O., Covaliu-Mierlă C.-I., Deleanu I.-M. (2023). From Plants to Wound Dressing and Transdermal Delivery of Bioactive Compounds. Plants.

[15] Elghandour M.M.M.Y., Maggiolino A., Vázquez-Mendoza P., Alvarado-Ramírez E.R., Cedillo-Monroy J., De Palo P., Salem A.Z.M. (2023). *Moringa oleifera* as a Natural Alternative for the Control of Gastrointestinal Parasites in Equines: A Review. Plants.

[16] Sehaki C., Jullian N., Ayati F., Fernane F., Gontier E. (2023). A Review of *Pistacia lentiscus* Polyphenols: Chemical Diversity and Pharmacological Activities. Plants.

